# Classification of viral zoonosis through receptor pattern analysis

**DOI:** 10.1186/1471-2105-12-96

**Published:** 2011-04-13

**Authors:** Se-Eun Bae, Hyeon Seok Son

**Affiliations:** 1 Laboratory of Computational Biology & Bioinformatics, Institute of Health and Environment, Graduate School of Public Health, Seoul National University, 599 Gwanak-ro, Gwanak-gu, Seoul 151-742, Korea; 2 Interdisciplinary Graduate Program in Bioinformatics, College of Natural Science, Seoul National University, 599 Gwanak-ro, Gwanak-gu, Seoul 151-742, Korea

## Abstract

**Background:**

Viral zoonosis, the transmission of a virus from its primary vertebrate reservoir species to humans, requires ubiquitous cellular proteins known as receptor proteins. Zoonosis can occur not only through direct transmission from vertebrates to humans, but also through intermediate reservoirs or other environmental factors. Viruses can be categorized according to genotype (ssDNA, dsDNA, ssRNA and dsRNA viruses). Among them, the RNA viruses exhibit particularly high mutation rates and are especially problematic for this reason. Most zoonotic viruses are RNA viruses that change their envelope proteins to facilitate binding to various receptors of host species. In this study, we sought to predict zoonotic propensity through the analysis of receptor characteristics. We hypothesized that the major barrier to interspecies virus transmission is that receptor sequences vary among species--in other words, that the specific amino acid sequence of the receptor determines the ability of the viral envelope protein to attach to the cell.

**Results:**

We analysed host-cell receptor sequences for their hydrophobicity/hydrophilicity characteristics. We then analysed these properties for similarities among receptors of different species and used a statistical discriminant analysis to predict the likelihood of transmission among species.

**Conclusions:**

This study is an attempt to predict zoonosis through simple computational analysis of receptor sequence differences. Our method may be useful in predicting the zoonotic potential of newly discovered viral strains.

## Background

Viral zoonosis, the transmission of a virus from its primary vertebrate reservoir species to humans, requires ubiquitous cellular proteins known as receptor proteins [1]. Zoonosis can occur not only through direct transmission, but also through intermediate reservoirs or other environmental factors [2-4]. The zoonotic viruses can be categorized according to genotype; of the various classes of viruses, the RNA viruses exhibit the highest mutation rates [5]. Most zoonotic viruses are RNA viruses that change their envelope proteins to facilitate binding to various receptors of host species [6,7]. The high mutation rate of envelope proteins [5] hinders the development of accurate vaccines, as does the great ability of the RNA viruses to infect host species in order to exploit host proteins for viral reproduction [8].

Lacking the ability to self-replicate, viruses must utilize the replication apparatus of their host cells [9]. Viral infection of a cell begins with attachment of the virus to the cell surface [6,10,11]. During attachment to the cell membrane, the viral envelope protein (a structural protein) interacts with the host-cell receptor protein(s) [12]. In non-envelope viruses, the capsid plays this role. The cell receptors that play a major role in viral attachment are predominantly membrane proteins of the immunoglobin superfamily [13-15]. The identification of virus-binding cellular receptors was rapidly accelerated in the late 1980s owing to developments in the use of monoclonal antibodies and molecular cloning techniques [15]. The various receptors that have been found are surface matrix structures containing carbohydrate, lipid, and protein moieties [1,16,17]. In some cases, viral attachment also exploits co-receptors. For example, HIV, which uses the CD4 molecule as its receptor, uses the CXCR4 and CCR5 co-receptors to strengthen the effectiveness of infection [1,14,18,19]. Similarly, hepatitis C virus utilizes CD81 as a receptor and LDLR as a co-receptor [20].

Since the host-cell range of a specific virus is predetermined by its ability to recognize specific receptors, the similarities between the receptors of its primary reservoir host cell and the potential human host cell play a major role in determining the likelihood of viral zoonosis. Here, we analysed zoonotic and non-zoonotic RNA viruses along with their cellular receptors in human and (non-human) primary reservoir species to extract the receptor characteristics common to zoonosis. Viruses not previously reported to infect humans were classified as non-zoonotic viruses. We excluded all viruses known to utilize co-receptors; *i.e.*, only virus-receptor interactions occurring through virus tropism and pathogenesis were considered [5,21]. The receptors and viruses examined in this study are listed in Table 1.

**Table 1:** Similarity scores of host receptor pairs.

Virus (receptor)	Host Species		^g^S_i,1_	^g^S_i,2_	^g^S_i,3_	g
Influenza A virus (NANA- synthase)	Gallus gallus^#^	Rattus norvegicus	0.810	0.841	0.853	1
	Gallus gallus^#^	Homo sapiens	0.855	0.912	0.861	1
	Rattus norvegicus	Homo sapiens	0.951	0.954	0.947	1

HIV (CD4)	Pan troglodytes^#^	Chlorocebus pygerythrus	0.919	0.925	0.899	1
	Pan troglodytes^#^	Homo sapiens	0.988	0.996	0.919	1
	Chlorocebus pygerythrus	Homo sapiens	0.905	0.975	0.794	1

FMDV (Integrin alpha V)	Sus scrofa^#^	Bos Taurus	0.964	0.976	0.859	1
	Sus scrofa^#^	Homo sapiens	0.949	0.978	0.951	1
	Bos Taurus	Homo sapiens	0.948	0.978	0.952	1

SARS (ACE2)	Felis catus^#^	Mustela putorius furo	0.855	0.950	0.897	1
	Felis catus^#^	Homo sapiens	0.790	0.936	0.852	1
	Mustela putorius furo	Homo sapiens	0.814	0.890	0.825	1

Hantavirus (Alpha (V) beta(3) integrin)	Mus musculus^#^	Rattus norvegicus	0.952	0.983	0.963	1
	Mus musculus^#^	Homo sapiens	0.867	0.951	0.906	1
	Rattus norvegicus	Homo sapiens	0.896	0.927	0.903	1

Rabies virus (AChR)	Canis lupus familiaris^#^	Homo sapiens	0.947	0.985	0.962	1
	Canis lupus familiaris^#^	Bos Taurus	0.280	0.373	0.366	2
	Bos Taurus	Homo sapiens	0.267	0.371	0.416	2

Enterovirus (CD55)	Sus scrofa^#^	Rattus norvegicus	0.238	0.392	0.287	2
	Sus scrofa^#^	Homo sapiens	0.309	0.432	0.354	2
	Sus scrofa^#^	Bos Taurus	0.440	0.371	0.406	2

TGE virus (APN)	Sus scrofa^#^	Epiphyas postvittana	0.276	0.294	0.241	2

Leukovirus (CAR1)	Gallus gallus^#^	Rattus norvegicus	0.120	0.118	0.138	2
	Gallus gallus^#^	Homo sapiens	0.092	0.108	0.146	2
	Gallus galllus^#^	Mus musculus	0.113	0.150	0.130	2

VSV (PS)	Culex quinquefasciatus^#^	Bos Taurus	0.570	0.733	0.480	3
	Culex quinquefasciatus^#^	Homo sapiens	0.461	0.537	0.523	3

Similarity scores (^g^S_i,1_, ^g^S_i,2_, ^g^S_i,3_) of host receptor pairs. The scores are calculated between pairs of species and at least one infected host is included in each pair. Groups represent infection (g = 1), non-infection (g = 2), and near-infection (g = 3) respectively. The primary reservoirs are designated as #. Abbreviation; NANA-synthase: N-acetyl neuraminic acid (Sialic acid) synthase, HIV: Human Immunodeficiency Virus, CD4: Cluster of differentiation 4, FMDV: Foot-and-Mouth disease virus, SARS: Severe Acute Respiratory Syndrome, ACE2: Amgoptemsom-Converting Enzyme 2, AChR: Acetylcholine receptor, CD55: Decay-accelerating factor, TGE virus: Transmissible Gastroenteritis virus, APN: Aminopeptidase N, CAR1 : Coxsackievirus-adenovirus receptor, VSV: Vesicular Stomatitis Virus, PS: Phosphatidyl serine

We hypothesized that the major barrier to the transmission of viruses between species is the difference in cellular receptor sequences. In other words, the specific amino acid sequence of the receptor should be the major determinant of the ability of the viral envelope protein to attach to the cell. Ordinary sequence alignment protocol tells us overall sequence similarity which we thought useful but insufficient because most receptors are membrane proteins and membrane proteins consist of distinctive hydrophobic and hydrophilic parts. Therefore, we analysed host-cell receptor sequences for their hydrophobicity/hydrophilicity characteristics. We then analysed these properties for similarities among receptors of different species to predict the likelihood of transmission across species, including humans. To our best knowledge, this study is the first attempt to predict zoonosis through a simple analysis of receptor sequence similarities and differences. This method may be useful in predicting the zoonotic potential of newly discovered viral strains.

## Results and Discussion

The pair-wise receptor sequence similarities (^g^S_i,1_, ^g^S_i,2_, and ^g^S_i,3_) between host-species pairs for each virus family are shown in Table 1. For logical comparisons, each virus contains at least one infected host (the primary reservoir, designated as "#" in Table 1). As shown in Table 1, the similarity scores for the infected group (g = 1) were high, ranging from 0.790 to 0.988 for ^1^S_i,1_, from 0.841 to 0.996 for ^1^S_i,2_, and 0.794 to 0.962 for ^1^S_i,3_. All pair-wise comparisons in group 1 (human vs. primary reservoir, primary reservoir vs. host, and human vs. host) yielded high similarity scores, indicating a high similarity among receptor sequences. The similarity scores were comparatively low in the non-infection group (g = 2), ranging from 0.092 to 0.440 for ^2^S_i,1_, from 0.108 to 0.432 for ^2^S_i,2_, and from 0.130 to 0.416 for ^2^S_i,3_. For group 2, both the primary host species and non-infected species are listed to illustrate the differences in similarity. In pair-wise comparisons, all the non-infection cases yielded low similarity values, *i.e.*, the receptor sequences differed significantly from each other.

We assume that a low similarity in receptor sequences disfavors infection despite the existence of a common receptor. For example, enterovirus infects only *Sus scrofa *(pig); it does not infect *Rattus norvegicus *(rat) or *Homo sapiens *(human) because of the high transmission barrier. Similarly, for leukovirus, only *Gallus gallus *(chicken) is infected as a primary reservoir; because of the high transmission barrier, *R. norvegicus *and *H. sapiens *are not infected. These results imply that for non-infection cases, species barriers exist, and the propensity to cross the barrier is determined by the sequence similarity between the potential and primary host receptors.

Similarity scores for rabies virus were low between *Canis lupus familiaris *(domestic dog) and *Bos Taurus *(domestic cow) (^2^S_i,1 _= 0.280, ^2^S_i,2 _= 0.373, and ^2^S_i,3 _= 0.366) and also between *B. taurus *and *H. sapiens *(^2^S_i,1 _= 0.267, ^2^S_i,2 _= 0.371, and ^2^S_i,3 _= 0.416) but were high between *C. l. familiaris *and *H. sapiens *(^1^S_i,1 _= 0.947, ^1^S_i,2 _= 0.985, and ^1^S_i,3 _= 0.962). Clearly, *C. l. familiaris *is the primary reservoir, and transmission of the disease to *H. sapiens *is possible only because of the high human/dog receptor similarity. Thus, for particular viruses, transmission of disease may be species-selective, although common receptors exist among species. Furthermore, infection specificity may be determined by the species barrier, which results from receptor differences.

**Figure 1:**
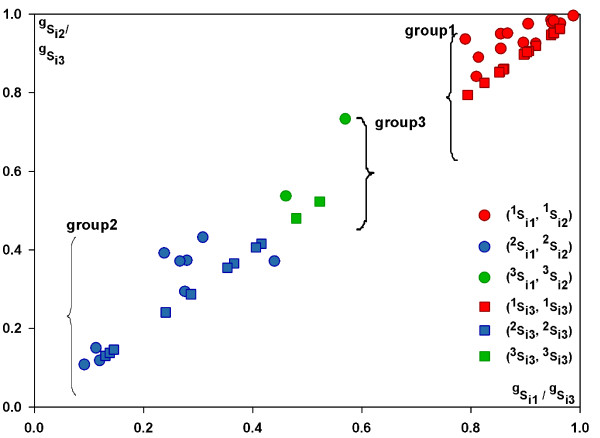
**Similarity scores of among groups**. Three kinds of pair-wise similarity scores (^g^S_i,1_, ^g^S_i,2_, ^g^S_i,3_) are plotted in two dimensional space to show clear differences among groups. Groups 1, 2 and 3 are each well separated; the results show clearly that the receptor sequences from cases of cross-species infection are well distinguished from those of other infection cases.

The values in Table 1 are plotted in Figure 1 to illustrate the differences among groups. The *x*- and *y*-axes denote ^g^S_i,1 _and ^g^S_i,2_, respectively, where "g" is the group classification. All pair-wise similarity scores are shown. Groups 1, 2 and 3 are each well separated in the colour-coded two-dimensional space. The results provide clear evidence that the receptor sequences from cases of cross-species infection are well separated from those of other infection cases. From these observations, we conclude that receptor differences are a major contributing factor to the potential of a specific viral strain to cross species barriers for transmission. In other words, the species dependence of infection is indirectly related to the receptor sequence similarity. This finding implies that once the receptor sequences of the primary reservoir and possible hosts are known, we might be able to predict the likelihood of viral disease transmission. The accuracy of these classifications can be judged by subsequent assessment of cases of actual zoonotic transmission to humans.

Our analysis revealed significant differences in receptor similarity between infection and non-infection cases. The similarity values, and the experimentally determined group categories were fed into a statistical discriminant analysis to logically predict infection (or zoonosis, in the case of human infection). As described in the Materials and Methods section, the values D_i_^2 ^(i = 1, 2, 3) were calculated from the data in the Table 1 to yield results of a specific discriminant analysis.

The statistical discriminant analysis was verified using a test set of four viruses that were deliberately excluded from the training set. The viruses whose groups were predicted using the discriminant analysis are shown in Table 2. The first virus, feline immunodeficiency virus (FIV), uses *Felis catus *(domestic cat) as its primary host and CD4 as its receptor. According to the literature [22,23], FIV infection of humans is rare but has been reported. Our method categorized this case as near-infection (G = 3). The second virus, classical swine fever virus, is known to be non-zoonotic and was classified as such by our method (G = 2). Thirdly, the encephalomyocarditis virus infects *S. scrofa *but has been known to cause sporadic infections in *H. sapiens*; it was classified as group 1 (G = 1) by our method. Finally, the Lass virus is known to be zoonotic and was classified as group 1 (G = 1) by our method.

**Table 2:** Virus group prediction.

Virus (receptor)	Host Species		S_1_	S_2_	S_3_	D_1_^2^	D_2_^2^	D_3_^2^	Pyyredicted group (G)
FIV (CD4)	Felis catus^#^	H.sapiens	0.289	0.671	0.530	289.991	204.386	3	3
CSFV (CD2)	Sus scrofa^#^	H.sapiens	0.285	0.299	0.465	242.079	2	169.443	2
EMCV (VCAM1)	Sus scrofa^#^	H.sapiens	0.737	0.779	0.728	1	138.266	44.413	1
Lassa (α dysglycan)	Mus musculus^#^	H.sapiens	0.956	0.909	0.935	1	292.616	17.074	1

Verification results using a test set of four viruses that were deliberately excluded from the training set. Abbreviation; FIV: Feline immunodeficiency virus, CSFV: Classic Swine Fever Virus, CD2: Cluster of differentiation 2, EMCV: Encephalomyocarditis virus, VCAM1: Vascular cell adhesion protein 1, Lassa: Lassa virus.

In Table 2, the hydrophilic similarity scores (S_1_) show less consistency, comparing to the hydrophobic scores (S2), with the predictive values (G). From the result, it could be said that the hydrophobic characteristics of receptor sequence might be the key contributor to the prediction. However, this observation should only be carefully interpreted because the variables (S1, S2, S3) are complementary in the statistical process.

## Conclusions

Our analysis of viral receptor sequences shows that the likelihood of viral infection correlates with the similarity in sequence of the primary and host receptors. This result is not surprising, because viral infection also inversely correlates with the inhibition of viral coat protein binding to the receptors. Importantly, we were able to establish this relationship at the amino acid sequence level, allowing for the prediction of possible human infection at an early stage of a viral outbreak, before the structures of viral coat proteins and receptors are known. Therefore, once the receptor sequences of primary reservoir and the potential host are known, the likelihood of viral infection can be predicted if the virus does not mutate too abruptly. Our simplistic approach needs further refinement because the complex processes of host tropism of viruses are largely ignored in our current method. For example, the process of host immune response could be included for better prediction of zoonosis. Although further refinements of our methods and analyses of larger databases are needed, this simple conceptual approach may be useful, even now, as a basic tool for the classification of zoonosis of new viral species.

## Methods

### Data collection

Viral infection requires the insertion of viral genes into host cells. Such a process begins with the binding of coat proteins to host receptors, and in some cases, co-receptors [24]. Ten RNA viruses (seven zoonotic viruses and three non-zoonotic viruses) were investigated. Viruses that use co-receptors were excluded from the study. Receptor sequence data for each virus were collected from the National Center for Biotechnology Information http://www.ncbi.nlm.nih.gov/, and the research literature was examined to determine the specific species tropism of each virus [[25], http://www.ictvonline.org/]. The viruses, host species, receptors, receptor sequences, and infection information for each host are shown in Table 1. We selected viruses that are each a representative of a different family, with different primary reservoirs. Viruses with unknown or poorly defined host receptors (particularly human receptors) were excluded from the study. Orthologues of the human receptor sequences for the non-zoonotic viruses were collected to allow for clear comparison with zoonosis cases.

### Discriminant analysis for data analysis

To calculate sequence similarities among host receptors for each virus, we first conducted a pair-wise sequence alignment using Clustal X [26,27]. We verified the alignment results with BLAST [28] and prank [[29], http://www.ebi.ac.uk/goldman-srv/prank/] and both alignment tools produced same reliable results as Clustal X. From the resulting alignment, we counted the numbers of matched amino acids and calculated three kinds of sequence similarity scores. The total sequence similarity scores were defined as:

and

where N_tot _is the total number of amino acids in one sequence string; n_tot _is the total number of matched amino acids in the sequence; N_phi _and N_pho _are the numbers of hydrophilic and hydrophobic amino acids in the sequence, respectively; N_others _is the number of deleted amino acids (gaps/insertions in sequence) plus the number of amino acids with undetermined properties; n_phi _and n_pho _are the numbers of hydrophilic and hydrophobic amino acids matched, respectively; and ^g^S_i,1 _is the similarity score for hydrophilic residues of the i^th ^row of infection group g. Here, there are only three groups: g = 1, 2, or 3, which are the infection, non-infection, and near-infection groups, respectively. The interspecies infection information was identified and classified among three infection states: group 1 (g = 1) represents infection; group 2 (g = 2) represents non-infection; and group 3 (g = 3) represents near-infection. By definition, if a group 1 species pair includes humans, then the infection is zoonotic. Decisions for grouping were made on the basis of experimental and epidemiological studies reported in the literature [4,30-33].

The variables (shown in Table 1) were arranged in matrices to allow for discriminant analysis, a method of multivariate analysis that can determine the group related to variables [34]. Each group has three columns and *l, m*, or *n *rows, depending on the numbers of variable sets. Here, the matrix for group 1 is defined as:

Similarly, ^2^S and ^3^S were defined as:

and

All of the related variables were tabulated as shown in Table 1. From the above matrices, three averages were found for each group:

The averages , , and  for group 2 and , , and  for group 3 were calculated similarly.

Three covariant matrices were constructed as:

where

and

where

and

Similar treatments yielded the ^2^C and ^3^C matrices, resulting in three covariance matrices (^1^C, ^2^C, and ^3^C). We then created a pool-within-class covariance matrix P. If we define *L = 3l-1*, *M = 3m-1*, and *N = 3n-1*, then:

where

also

We next found the inverse matrix **I**, where **I = P^-1^**. Because there were three groups in our study, we predicted the likelihood of infection for a virus of unknown infection condition by calculating the Mahalanobis distance (generally D^2 ^= d_1 _× C^-1 ^× D_i_).

Here, expansion of D^2 ^yielded three equations:

where

where *S_1_*, *S_2_*, and *S_3 _*are the input variables; here, they were similarity variables of a virus of an unknown infection group.

Group classification (G) was identified using the criterion:

For example, if D_1_^2 ^is the minimum among three values from the above set of three equations, then G = 1; *i.e.*, "group 1" is the group classification. To automate the mathematical process described above, we developed a Java computer program named ZOO. To evaluate the accuracy of our method and software, we analysed a test data set (described in the Results & Discussion section).

## Authors' contributions

SEB and HSS have developed the methods and have conducted subsequent data analysis. Both authors have drafted, read and approved the manuscript.
